# The Social Responsibility of Universities in Promoting Senior Universities – From a Sociological Perspective

**DOI:** 10.1093/geroni/igaf122.3171

**Published:** 2025-12-31

**Authors:** Weng Pi Ching

**Affiliations:** National Chung Cheng University, Minxiong, Chiayi, Taiwan (Republic of China)

## Abstract

In 2025, Taiwan has officially entered a super-aged society. The social responsibility of universities extends beyond providing formal education to young students; they also play a crucial role in supporting and promoting senior universities. Since 2010, Taiwan’s Ministry of Education has sought contractors through a bidding process to implement senior universities. Currently, 83 universities in Taiwan operate senior universities, with 3,500 elderly students enrolled, fulfilling their dreams of attending university. However, more than 10,000 elderly individuals are still eager to join. This study employs in-depth interviews and survey methods. From a sociological perspective, interviews were conducted with the project director and an assistant. From the perspective of resource allocation, interviews were held with personnel responsible for program implementation at eight universities across Taiwan, from north to south. From the standpoint of Symbolic Interactionism, 30 elderly students participating in senior universities were interviewed, followed by a questionnaire survey. The study concludes that the project director aims to uphold social justice and equal opportunity through the implementation of senior universities. Program coordinators utilize university-specific resources to design courses, which account for 40% of the entire curriculum, ensuring that educational funding is used effectively. Elderly students, despite not earning a formal degree, construct their self-identity through interactions, pursuing their studies based on personal interests. They find joy in learning at senior universities, establish positive interpersonal relationships, and ultimately fulfill their dream of attending university. These valuable experiences can serve as a reference for other countries.

